# Building malaria out: improving health in the home

**DOI:** 10.1186/s12936-016-1349-8

**Published:** 2016-06-15

**Authors:** Lucy S. Tusting, Barbara Willey, Jo Lines

**Affiliations:** Big Data Institute, Nuffield Department of Medicine, University of Oxford, c/o Wellcome Trust Centre for Human Genetics, Roosevelt Drive, Oxford, OX3 7BN UK; Department of Infectious Disease Epidemiology, London School of Hygiene and Tropical Medicine, Keppel Street, London, WC1E 7HT UK; Department of Disease Control, London School of Hygiene and Tropical Medicine, Keppel Street, London, WC1E 7HT UK

**Keywords:** Malaria, *Plasmodium*, *Anopheles*, Mosquito, Disease vector, Housing, Eaves

## Abstract

Malaria prevalence has halved in endemic Africa since 2000, largely driven by the concerted international control effort. To achieve the new global targets for malaria control and elimination by 2030, and to sustain elimination once achieved, additional vector control interventions are urgently needed to supplement long‐lasting insecticide-treated nets and indoor residual spraying, which both rely on effective insecticides for optimal protection. Improving housing and the built environment is a promising strategy to address this need, with an expanding body of evidence that simple modifications to reduce house entry by malaria vectors, such as closing eaves and screening doors and windows, can help protect residents from malaria. However, numerous questions remain unanswered, from basic science relating to the optimal design of house improvements through to their translation into operational use. The *Malaria Journal* thematic series on ‘housing and malaria’ collates articles that contribute to the evidence base on approaches for improving housing to reduce domestic malaria transmission.

## The need for supplementary malaria control interventions

Unprecedented investment in malaria control and elimination has led to a halving of malaria prevalence in endemic Africa since 2000 [1]. Despite this progress, the disease remains intractable in many settings and a major source of morbidity and mortality worldwide. Ambitious new targets aim to reduce malaria case incidence and mortality by a further 90 % globally and to eliminate malaria in 35 endemic countries during 2016–2030 [2].

Long‐lasting insecticide-treated nets (LLINs) and indoor residual spraying (IRS) will certainly be needed to achieve elimination. However, to reach and stabilize the state of elimination in Africa, strategies are also needed to reduce transmission in the long term, preferably without insecticides [3].

Reliance on a small group of insecticides and anti-malarial drugs has inevitably imposed massive pressure on vector and parasite populations, contributing to the widespread emergence of *Anopheles* resistant to pyrethroids across Africa [4] and parasites resistant to artemisinin in South East Asia [5]. Continuing effective treatment will require appropriate use of existing anti-malarials as well as new combinations and new drugs. For vector control, alternative insecticide compounds are needed in the short run, but in the long run there should be less reliance on chemical-based intervention. It is well-established that malaria is both a cause and a consequence of underdevelopment, because of its intricate connections with the physical and social environment [6]. This is a challenge, but it is also an opportunity: it means that processes of economic, social and environmental development can be harnessed and used to “build malaria out”.

To put this into practice, there is a need to explore opportunities to supplement existing malaria control interventions with alternative strategies that offer protection across all transmission settings and can sustainably prevent reintroduction post-elimination. Within endemic countries, it is acknowledged that tackling malaria requires the participation of all branches of government (not just the Ministry of Health) and that malaria control must be linked with plans for economic development. The multisectoral nature of the task has been explained by the Roll Back Malaria Partnership [7], incorporated into its implementation plans for 2016–2030 [8] and outlined within the World Health Organization’s initiative for Integrated Vector Management, which is an evidence-based, adaptive and multisectoral approach to vector control [9]. The recognition that malaria needs a more integrated approach reflects the shift of perspective expressed in the Sustainable Development Goals, which emphasize the links between health and environment [10].

## The potential of improved housing

Improving housing and the built environment is a promising means to support a more integrated and sustainable approach to malaria across the whole spectrum of endemicity. *Anopheles* mosquitoes bite during the night, and the species that transmit malaria in Africa bite mainly between 10 p.m. and 4 a.m., when most people are indoors. Therefore, structural modifications that reduce house entry by mosquitoes can help to reduce human exposure to infectious bites. Such features may include closed eaves (the gap between the top of the wall and bottom of the roof), screened doors, and windows and the presence of a ceiling [11–14]. Other mechanisms may be involved; for example, houses constructed with metal roofs may be less attractive as indoor resting places for normally endophilic mosquitoes [15]. As countries develop and disposable incomes increase, many such incremental house improvements are visibly occurring across the endemic world [13, 15, 16].

Following decades of relative neglect as a malaria intervention, the epidemiological evidence base for improved housing is far smaller than for primary malaria interventions. Notions of “building out” malaria, first proposed by Celli [17] and Manson [18], became increasingly overlooked following the development of DDT and IRS [19]. To date, only one randomized controlled trial (RCT), conducted in The Gambia, has evaluated a house screening intervention against malaria and measured epidemiological outcomes [20]. Indeed, a recent review of housing and malaria highlighted the absence of data from many geographical regions, the paucity of intervention studies and the high risk of bias within and across studies [15].

Despite the gaps in the evidence, improved housing shows promise for reducing malaria transmission. In The Gambia, full house screening (with netting-covered doors and windows, screened ceilings and blocked eaves) reduced the prevalence of anaemia in children by 47 % [20]. In a recent systematic review and meta-analysis, residents of ‘modern’ houses were observed to have a 47 % lower odds of malaria infection and a 45–65 % lower odds of clinical malaria, compared to residents of ‘traditional’ houses in settings across Africa, Asia and Latin America [15]. Though the quality of the evidence was judged to be low, the direction and consistency of effects indicated that housing may be an important risk factor for malaria. This association has been observed at both extremes of the transmission spectrum, from Swaziland to Uganda [14].

## Where to from here?

Key questions must be tackled if malaria control is to be supplemented with better housing [21]. These fall into four themes. First, there are basic science questions on housing interventions themselves, including what features are effective, what packages of house improvements are sufficient in different eco-epidemiological settings and their associated effectiveness. Effectiveness must be demonstrated against both entomological and epidemiological outcomes and in the context of existing interventions. Second, there are questions of safety and unintended consequences, including potentially adverse effects of interventions that reduce indoor ventilation on the risk of respiratory disease and potentially beneficial effects on nuisance biting and the transmission of other vector-borne disease. Third, the acceptability of housing interventions and their interaction with education, the use of other control measures and health-seeking behaviour must be understood. Fourth, there are critical questions on implementation, relating to cost, short- and long-term cost-effectiveness compared to conventional interventions, funding mechanisms, scale-up, sustainability and long-term maintenance and repair in both rural and urban areas.

In response to many of these questions, a promising research pipeline is emerging. For example, a second household cluster RCT is underway in The Gambia to determine whether modern housing provides incremental protection against clinical malaria over current best practice of LLINs and prompt treatment [22]. Field studies on ‘eave tubes’ treated with resistance-breaking actives are gaining momentum [23, 24] and other house-based malaria interventions, such as push–pull systems [25] and portable housing for mobile workers [26], continue to develop. Such research should be considered central to strengthening future malaria control and elimination efforts.

To provide a new forum for research on housing and malaria, this thematic series on ‘housing and malaria’ invites articles, reviews, and commentaries that contribute to the evidence base on approaches for improving housing to reduce domestic malaria transmission. The aim is to provide a platform to encourage interdisciplinary thinking; to collate evidence, old and new; and to stimulate discussion. Contributions from all disciplines are welcomed.

## Abbreviations

DDT: dichlorodiphenyltrichloroethane
IRS: indoor residual spraying
LLINs: long-lasting insecticide-treated nets
RCT: randomized controlled trial
